# Nitric oxide inhibition ameliorates cortical proteomic changes in the *Cntnap2*^-/-^ and *Shank3*^Δ4–22^ mouse models of autism spectrum disorder

**DOI:** 10.1186/s13229-026-00716-1

**Published:** 2026-04-20

**Authors:** Wisam Bazbaz, Maryam Kartawy, Igor Khaliulin, Haitham Amal

**Affiliations:** 1 Institute for Drug Research, School of Pharmacy, Faculty of Medicine, The Hebrew University of Jerusalem, Jerusalem, Israel; 2 Rosamund Stone Zander and Hansjoerg Wyss Translational Neuroscience Center, Boston Children’s Hospital, Harvard Medical School, Harvard University, Boston, MA USA

**Keywords:** Autism spectrum disorder, Behavior, Synapse, Nitric oxide, Neuronal nitric oxide synthase (nNOS), *Cntnap2*, *Shank3*, Mouse models, Mass spectrometry, Systems biology

## Abstract

**Background:**

Autism spectrum disorder (ASD) is a neurodevelopmental disorder with a strong genetic component, and over a thousand associated genes have been identified, including *CNTNAP2* and *SHANK3*. Our previous work using *Cntnap2*^-/-^ and *Shank3*^Δ4–22^ ASD mouse models implicated dysregulated nitric oxide (NO) signaling in ASD-related behaviors, which were improved by inhibition of neuronal nitric oxide synthase (nNOS) with 7-Nitroindazole (7-NI). However, the molecular mechanisms linking NO signaling to ASD pathology remain poorly defined.

**Methods:**

We performed mass spectrometry-based global proteomic profiling of cortical tissue from both mouse models under baseline conditions and following 7-NI treatment. Systems biology and bioinformatics analyses were used to identify differentially expressed proteins, enriched pathways, and treatment-responsive networks. Cross-model comparisons were performed to assess molecular convergence and overlap with human ASD-risk genes. Behavioral and biochemical assessments were reanalyzed to evaluate ASD-like phenotypes and treatment effects.

**Results:**

Treatment with 7-NI improved ASD-like behavioral deficits in *Cntnap2* and *Shank3* mutant mice, including increased sociability and reduced anxiety-like behavior. 7-NI was also associated with attenuation of cortical protein alterations across synaptic, neuronal, and metabolic pathways, shifting subsets of dysregulated proteins toward wild-type expression levels. Despite distinct genetic mutations, the two models converged at the protein and pathway levels, including treatment-responsive proteins encoded by high-confidence human ASD risk genes.

**Limitations:**

Analyses were restricted to cortical tissue; additional brain regions may reveal complementary mechanisms. Mass spectrometry may underrepresent low-abundance proteins; larger sample sizes could improve statistical power. Potential off-target effects of 7-NI should also be considered.

**Conclusions:**

These findings show that nNOS inhibition improves ASD-like behaviors and is associated with partial normalization of altered cortical proteins across two genetically distinct ASD mouse models that display convergent molecular changes, including proteins encoded by high-confidence ASD risk genes.

**Supplementary Information:**

The online version contains supplementary material available at 10.1186/s13229-026-00716-1.

## Background

Autism spectrum disorder (ASD) is a highly heritable and heterogeneous neurodevelopmental disorder [1], characterized by impaired social communication, restricted interests, and repetitive behaviors. ASD affects approximately 1% of the global population [2]. Despite extensive research, the exact cause of ASD remains incompletely understood [3]. Nevertheless, ASD has a strong genetic basis, with over a thousand risk genes associated with the disorder [4], including high-risk ASD genes such as contactin-associated protein-like 2 (*CNTNAP2*) [5, 6] and SH3 and multiple ankyrin repeat domains 3 (*SHANK3*) [7–9]. 

*CNTNAP2* encodes a presynaptic transmembrane protein called CASPR2. Studies suggest that CASPR2 plays a role in neuronal migration, myelination, and neurotransmission, impacting both inhibitory γ-aminobutyric acid (GABA)ergic and excitatory glutamatergic neuronal functions [10]. 

*SHANK3* encodes a scaffolding protein located in the postsynaptic density of excitatory synapses [11]. SHANK3 plays an important role in synapse formation, maturation, development, and maintenance [12]. Additionally, more than 1% of ASD cases involve a mutation in this gene [13].

We previously showed that *Cntnap2*^-/-^ and *Shank3*^*Δ4*–22^ (hereafter referred to as *Cntnap2* and *Shank3*) mouse models display several ASD-like phenotypes, including reduced sociability, impaired social novelty, decreased novelty-seeking, elevated anxiety, and repetitive behaviors, in addition to several alterations in synaptic protein expression and dendritic spine density [14–16]. These previous studies revealed that nitric oxide (NO) and NO-mediated post-translational modifications, particularly S-Nitrosylation (SNO), may act as crucial contributors to the molecular alterations observed in ASD and other neurological disorders [17–20].

NO is a small, highly diffusible gaseous radical that functions as both a secondary messenger [21] and a neurotransmitter [22]. It is produced by nitric oxide synthase (NOS), which converts L-arginine to L-citrulline and NO [23]. NO regulates various physiological and pathophysiological processes including vascular homeostasis [24], neurotransmission, inflammatory reactions, and immune responses [25–27].

In earlier works, we were able to reduce NO production in the *Cntnap2* and *Shank3* mutants by inhibiting neuronal nitric oxide synthase (nNOS) with 7-Nitroindazole (7-NI), leading to amelioration of the ASD-like behavioral phenotypes in both models [14, 16]. 7-NI is a relatively selective nNOS inhibitor [28–30]. It inhibits nNOS by competing with L-arginine, thereby reducing NO production [31]. Its small, lipophilic nature allows it to cross the blood-brain barrier via passive diffusion [32], making it effective for targeting the brain.

NO and SNO signaling are implicated in ASD pathology but remain poorly mechanistically understood. Here, we applied mass spectrometry-based proteomics integrated with bioinformatics and systems biology analyses to define cortical protein alterations and identify the molecular mechanisms underlying the rescue effects of nNOS inhibition by 7-NI. We compared *Cntnap2* and *Shank3*, which exhibit similar ASD-like phenotypes despite distinct presynaptic and postsynaptic mechanisms, to test whether shared molecular pathways underlie these behaviors and whether nNOS inhibition produces convergent rather than model-specific effects.

## Methods

### Materials

Primary antibody against γ-aminobutyric acid (GABA) type A receptor β2 subunit (GABRB2; MA5-35130) was obtained from Thermo Fisher Scientific (Waltham, MA, USA). Anti–glutamate receptor 3 (GluA3; MAB5416) was purchased from Sigma-Aldrich (St. Louis, MO, USA). Anti–tropomyosin receptor kinase B (TrkB; ab322464) was obtained from Abcam (Cambridge, UK). Secondary antibodies including horseradish peroxidase (HRP)–conjugated anti-rabbit (#7076S) and HRP-conjugated anti-mouse (#7074S), and the protease and phosphatase inhibitor cocktail (#5872), were purchased from Cell Signaling Technology (Danvers, MA, USA). All other reagents were obtained from Sigma-Aldrich (St. Louis, MO, USA) or Bio-Rad Laboratories (Hercules, CA, USA).

#### Animals and mouse models

*Cntnap2* (Strain #7482), *Shank3* (Strain #2169), and C57BL/6J (Strain #:000664) mice were purchased from the Jackson Laboratory (Farmington, CT, USA). Mating pairs of homozygous *Cntnap2* mice were used for the *Cntnap2* mouse model of autism spectrum disorder (ASD). C57BL/6J mice were employed as a wild-type (WT) control group for *Cntnap2* mice. Heterozygous male *Shank3* mice were mated with heterozygous female *Shank3* mice, resulting in three genotypes: WT, heterozygous, and homozygous *Shank3* mice. WT littermates were used as controls for the *Shank3* mice. 6-week-old male *Cntnap2* and *Shank3* mice and their respective WT littermates were used in this study.

#### Pharmacological intervention and experimental groups

7-Nitroindazole (7-NI), a selective inhibitor of neuronal nitric oxide synthase (nNOS) [33], was dissolved in 1% dimethyl sulfoxide (DMSO) and then in corn oil. The mice received either an 80 mg/kg dose of 7-NI or a vehicle (1% DMSO in corn oil) via an intraperitoneal (IP) injection daily for 10 days. Six groups of mice were tested: (1) WT littermates of *Cntnap2* mice; (2) *Cntnap2* mice; (3) *Cntnap2* mice treated with 7-NI (*Cntnap2 +* 7-NI); (4) WT littermates of *Shank3* mice; (5) *Shank3* homozygous mice; and (6) *Shank3* homozygous mice treated with 7-NI (*Shank3* + 7-NI).

**Fig. 1:**
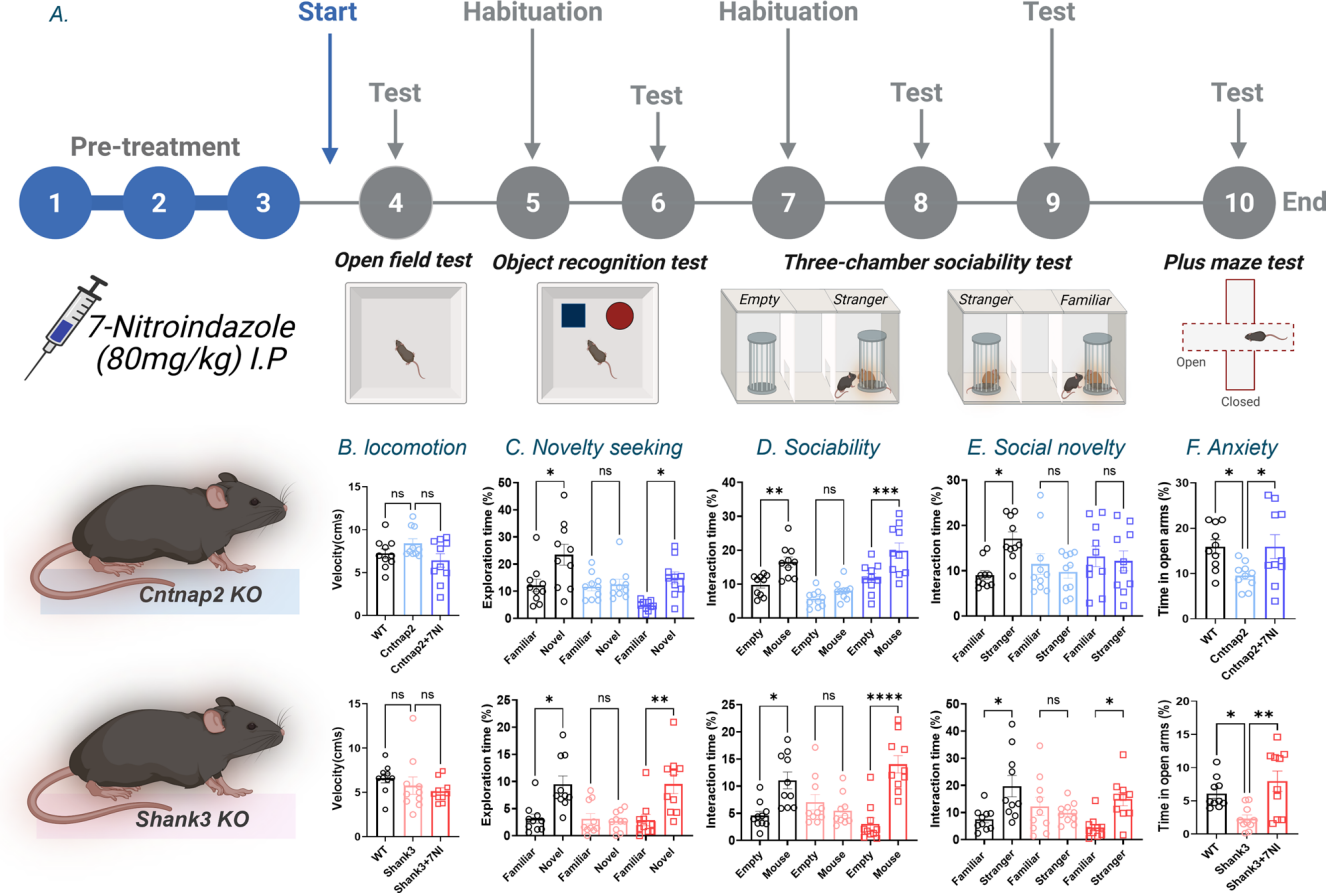
7-Nitroindazole treatment alleviates ASD-like phenotypes in *Cntnap2* and *Shank3* mouse models. (**A**) Behavioral timeline illustrating the injection schedule and the order of behavioral testing. Behavioral analyses were performed in the *Cntnap2* cohort (WT, *Cntnap2*, and *Cntnap2* + 7-Nitroindazole (7-NI); upper panels) and the *Shank3* cohort (*Shank3*-WT, *Shank3*, and *Shank3* + 7-NI; lower panels). n = 10 mice per group. (**B**) Open field test assessing locomotor velocity. (**C**) Novel object recognition test showing exploration time of familiar versus novel objects. (**D**) Three-chamber social interaction test (sociability) showing time spent in the mouse chamber versus the empty chamber. (**E**) Three-chamber social interaction test (social novelty) showing time spent interacting with a familiar versus a stranger mouse. (**F**) Elevated plus maze showing time spent in the open arms. One-way ANOVA followed by Dunnett’s multiple-comparisons test was used for the open field and elevated plus maze tests. Two-way ANOVA followed by Tukey’s multiple-comparisons test was used for the novel object recognition, sociability, and social novelty tests. ns, not significant; *, p < 0.05; **, p < 0.01; ***, p < 0.001; ****, p < 0.0001

#### Behavioral tests

Ten mice were randomly selected from a pre-existing dataset comprising both published and unpublished data and analyzed as a representative subset [14, 15]. Behavioral data were blindly reanalyzed; no new behavioral experiments were performed. Sample selection occurred prior to analysis and without knowledge of group outcomes. All experimental conditions (housing, handling, testing order, and time of day) were identical across groups.

All behavioral experiments were recorded and automatically analyzed using EthoVision XT 16 (Noldus Information Technology BV) with AI-assisted tracking of mouse position and body points. Interaction analyses were based on nose tracking, with identical software settings applied across all mice. Arenas and objects were cleaned with 5% Virusolve or 70% ethanol between trials and tests, as previously described [14–16, 34]. Mice received treatment for 10 consecutive days. Behavioral testing began on day 4 of treatment and was conducted sequentially in a fixed order as shown in Fig. 1A.

Open Field Test (Day 4):

Locomotor activity was assessed using the open field test in a white plastic arena (60 × 60 cm), with mean velocity (cm/s) recorded for 5 min.

Novel Object Recognition (Days 5–6):

Recognition memory was assessed using the novel object recognition test. Mice were habituated to the empty arena (open field arena) 24 h before testing. During the familiarization session, mice explored two identical objects for 5 min; 24 h later, one object was replaced with a novel object, and exploration time was recorded for 5 min. Exploration was defined as nose-oriented contact (≤ 1 cm).

Three-Chamber Social Test (Days 7–9):

Sociability and social novelty were assessed using the three-chamber test. Mice underwent a 5-min habituation; 24 h later, the mice underwent a 10-min sociability test with an unfamiliar, age- and sex-matched mouse versus an empty chamber. Social novelty was then assessed 24 h later by introducing a second unfamiliar mouse in place of the empty chamber, and exploration of familiar versus novel mice was recorded for 10 min.

Elevated Plus Maze (Day 10):

Anxiety-like behavior was assessed using an elevated plus maze (two open and two closed arms; 30 × 5 cm, 45 cm above floor). Mice were allowed to explore for 10 min, and time spent in open and closed arms was recorded.

A full description of the tests is provided in our previous publications [15, 16, 34].

#### Sample preparation for mass spectrometry

Four mice were used per group in the *Cntnap2* cohort, and three mice were used per group in the *Shank3* cohort. Cortical tissues were lysed in 5% sodium dodecyl sulfate (SDS)/50 mM Tris(hydroxymethyl) aminomethane hydrochloride (Tris HCl), and protein concentrations were measured using bicinchoninic acid (BCA) assay. Proteins (110 µg) were reduced with dithiothreitol, alkylated with iodoacetamide, and loaded onto S-Trap minicolumns for trypsin digestion. Peptides were eluted in three steps, pooled, dried, and stored at − 80 °C until further analysis. For full methodological details, see our previous study [35].

**Fig. 2:**
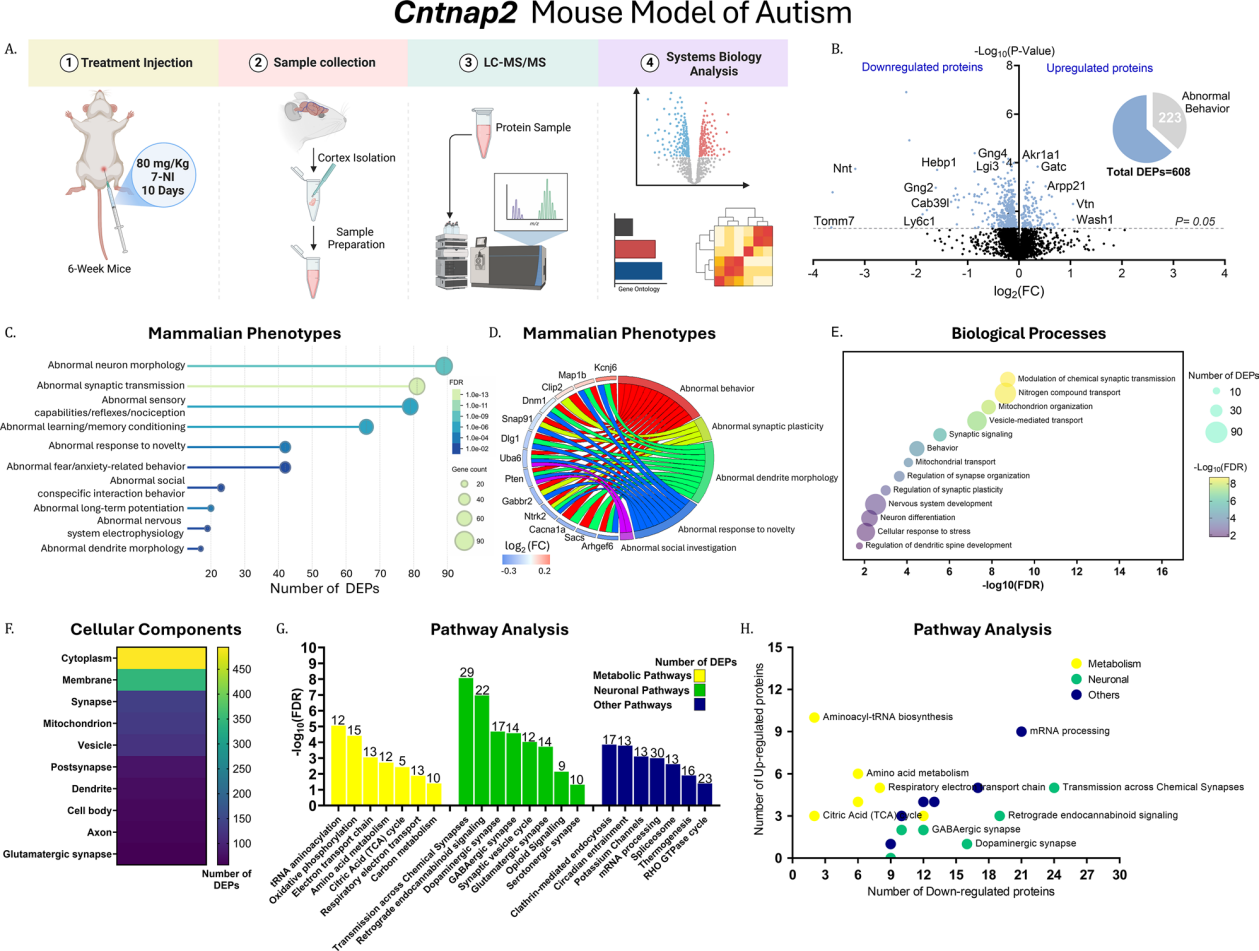
Quantitative analysis of differentially expressed proteins in the cortex of *Cntnap2* mice. (**A**) Graphical representation of the experimental workflow (*Cntnap2* cohort, n = 4; *Shank3* cohort, n = 3). (**B**) Volcano plot of differentially expressed proteins (DEPs) in the cortex of *Cntnap2*/WT mice. The x-axis shows log₂(fold change, FC) (upregulated proteins on the right, downregulated on the left), and the y-axis shows − log₁₀(P-value). The horizontal dashed line indicates the significance threshold (p = 0.05). The pie chart summarizes the number of DEPs associated with abnormal behavior. (**C**–**H**) Gene Ontology (GO) analysis of DEPs identified in *Cntnap2* mice: (**C**) Dot plot for mammalian phenotypes (color scale represents the number of DEPs), (**D**) GOChord diagram for mammalian phenotypes (color scale represents log₂(FC)), (**E**) Bubble plot for biological processes (color scale represents −log₁₀(false discovery rate, FDR)), (**F**) Heatmap for cellular components (color scale represents the number of DEPs), (**G**) Bar graph for pathways (y-axis represents −log₁₀(FDR); numbers on the bars indicate the number of DEPs per enriched term), and (**H**) Bubble plot for pathways (x-axis: number of downregulated DEPs; y-axis: number of upregulated DEPs). Metabolism-related pathways are shown in yellow, neuronal pathways in green, and other pathways in blue

#### Liquid chromatography (LC)

Liquid Chromatography-Mass Spectrometry (LC/MS) grade solvents were used for all chromatographic steps. Each sample was loaded using split-less nano-Ultra Performance LC (10 kpsi nanoAcquity; Waters, Milford, MA, USA). The mobile phase was (A) H_2_O + 0.1% formic acid and (B) acetonitrile + 0.1% formic acid. Sample desalting was performed online using a reversed-phase Symmetry C18 trapping column (180 μm internal diameter, 20 mm length, 5 μm particle size; Waters). The peptides were then separated using a T3 HSS nano-column (75 μm internal diameter, 250 mm length, 1.8 μm particle size; Waters) at a 0.35 µL/min rate. Peptides were eluted from the column into the mass spectrometer using a gradient of 4% to 30% of B. For full methodological details, see our previous study [35].

#### Mass spectrometry (MS)

Peptides were analyzed by nanoUPLC coupled to a quadrupole-orbitrap mass spectrometer (Q Exactive HFX or Fusion Lumos) in data-dependent acquisition mode. MS1 scans were acquired at high resolution over 375–1650 m/z, followed by MS2 scans of the top precursors with dynamic exclusion. Detailed instrument settings and acquisition parameters are described in our previous study [35].

#### Proteomics data processing

Raw data were processed with MaxQuant v1.6.6.0 or v2.0.1.0 software. The data were searched with the Andromeda search engine against the Uniprot murine proteome database (https://www.uniprot.org/) appended with common lab protein contaminants and the following modifications: Carbamidomethylation of C as a fixed modification, oxidation of M and protein N-terminal acetylation as variable modifications. Quantification was performed using the embedded FlashLFQ and protein inference algorithms. The Label-Free Quantification (LFQ) intensities were calculated and used for further calculations using Perseus v1.6.2.3 [36]. Decoy hits, as well as proteins that were identified based on a modified peptide only, were filtered out. The LFQ intensities were log-transformed, and only proteins with at least two valid values in at least one experimental group were kept. The remaining missing values were imputed.

#### Western blotting

Cortical tissues (*n* = 3 per group) were homogenized on ice in freshly prepared Radioimmunoprecipitation Assay buffer (RIPA buffer) containing 30 mM 4-(2-hydroxyethyl)-1-piperazineethanesulfonic acid (HEPES, pH 7.4), 150 mM sodium chloride (NaCl), 1% Nonidet P-40, 0.5% sodium deoxycholate, 0.1% SDS, 5 mM ethylenediaminetetraacetic acid (EDTA), 1 mM sodium orthovanadate (Na₃VO₄), 50 mM sodium fluoride (NaF), 1 mM phenylmethylsulfonyl fluoride (PMSF), and protease and phosphatase inhibitor cocktails (pH 7.7), using a Teflon pestle and Jumbo Stirrer (Thermo Fisher Scientific). Homogenates were centrifuged at 17,000 × g for 30 min at 4 °C, and protein concentrations were determined using the bicinchoninic acid (BCA) protein assay.

Equal amounts of protein were separated by sodium dodecyl sulfate–polyacrylamide gel electrophoresis (SDS-PAGE) and transferred by wet transfer onto polyvinylidene fluoride (PVDF) membranes (Bio-Rad). Membranes were blocked for 2 h at room temperature in Tris-buffered saline with Tween-20 (TBST) containing 5% dried skimmed milk. Membranes were incubated overnight at 4 °C with primary antibodies against GluA3, GABRB2, and TrkB (1:1000).

Following TBST washes, membranes were incubated with horseradish peroxidase-conjugated secondary antibodies for 2 h at room temperature. Protein bands were detected using enhanced chemiluminescence (ECL) and visualized with the Bio-Rad ChemiDoc imaging system. Band intensities were quantified using Bio-Rad Image Lab software and normalized to total lane protein, determined by stain-free imaging of the same membrane. Post-transfer stain-free imaging was performed on a subset of blots solely to validate transfer efficiency and uniformity.

#### Bioinformatics and statistical analysis

Behavioral data were analyzed using tests as specified for each assay. Open field and elevated plus maze data were analyzed using one-way analysis of variance (ANOVA) followed by Dunnett’s multiple comparisons test. Novel object recognition, sociability, and social novelty were analyzed using two-way ANOVA, with phenotype/treatment and stimulus (object or social target) as factors, followed by Tukey’s post hoc test for multiple comparisons. Proteomics analyses were performed using a Student’s t-test, applied after logarithmic transformation.

Raw p-values (*p* < 0.05) were used to define differentially expressed proteins (DEPs) for exploratory analyses, while false discovery rate (FDR)-adjusted p-values were calculated using the Benjamini–Hochberg procedure [37] and reported to account for multiple testing and ensure transparency. Effect sizes were estimated using Cohen’s d to quantify the magnitude of pairwise group differences [38], and 95% confidence intervals (CI95) were used to estimate the precision of these effects [39].

Raw p-values, FDR-adjusted p-values, Cohen’s *d*, and CI95 values calculated across all proteins and all experimental groups are provided in Supplementary Files 1 and 2.

For comparative analysis between models, the Jaccard index was calculated as the number of shared DEPs divided by the total unique DEPs. Information on high-risk ASD genes was obtained from the SFARI database (1,255 high-risk ASD genes, December 2025) [40], and the overlap with our altered proteins was assessed using a hypergeometric test [41]. Calculations and validations are provided in Supplementary File 4.

Gene ontology (GO) analysis of biological processes (BP), cellular components (CC), signaling pathways, mammalian phenotype annotations, and clustering (K-means/Markov cluster algorithm (MCL)) were performed using Search tool for the retrieval of interacting genes/proteins (STRING) database v11.5 [42, 43]; unconnected nodes were omitted when necessary. The −log_10_(FDR) transformation was applied, and fold changes (FC) were calculated as the log_2_ ratio of protein abundance between groups (e.g., WT vs. *Cntnap2*).

Protein interaction networks were visualized in Cytoscape v3.10.4 with an interaction score threshold of 0.4. Bar graphs, bubble plots, scatter plots, heatmaps, and volcano plots were generated using GraphPad Prism v10. GOChord plots were generated using GOplot in R [44], and Venn diagrams were created using Canva [45]. Human orthologs of mouse genes were identified using BioMart [46]. Adult brain expression was assessed using GTEx bulk RNA-sequencing data [47], and developmental expression from prenatal stages through adulthood was examined using BrainSpan (genes lacking orthologs were excluded) [48]. Illustrations were created using Biorender [49].

Western blot (WB) quantifications were analyzed using one-way ANOVA followed by Dunnett’s multiple comparisons test. Each lane represented one biological replicate; exact *n* values are reported in the figure legends.

## Results

### Behavioral assessment of *Cntnap2 * and *Shank3* autism mouse models following 7-Nitroindazole treatment

A series of behavioral tests were conducted to assess the effect of the neuronal nitric oxide synthase (nNOS) inhibitor 7-Nitroindazole (7-NI) on autism spectrum disorder (ASD)-like phenotypes in *Cntnap2* and *Shank3* mouse models (Fig. 1A).

In the open field test, locomotor activity was quantified as mean movement velocity, and no differences were observed between the groups in either model (Fig. 1B).

In the novel object recognition test, wild-type (WT) mice preferred the novel object, a preference absent in both mutants but restored by 7-NI treatment in *Cntnap2* and *Shank3* mice (Fig. 1C).

In the three-chamber sociability test, WT mice preferred the social partner, whereas both mutants showed no preference; 7-NI treatment restored social interaction to levels closer to WT in both models (Fig. 1D). In the social novelty test, WT mice preferred the novel mouse, whereas mutants did not. This deficit was not improved by 7-NI in *Cntnap2* mice but was restored in *Shank3* mutants (Fig. 1E).

In the elevated plus maze, mutants spent less time in the open arms than WT mice, whereas 7-NI treatment increased open-arm exploration to levels closer to WT in both models (Fig. 1F).

### Proteomics analysis of differentially expressed proteins in the cortex of *Cntnap2 *mice

High-throughput mass spectrometry (MS) identified 4,823 proteins in the cortex of *Cntnap2* mice, of which 608 were differentially expressed proteins (DEPs) (Figs. 2A & 2B). These DEPs were visualized using a volcano plot and included proteins involved in neurotransmission and signaling, such as the sodium-dependent noradrenaline transporter (NET; *Slc6a2*), excitatory amino acid transporter 1 (EAAT1; *Slc1a3*), aminoacylase-1 (ACY1; *Acy1*), and the soluble guanylate cyclase β1 subunit (sGCβ1; *Gucy1b1*).

Mammalian phenotype ontology analysis revealed significant enrichment of ASD-associated phenotypes, including abnormal behavior (223 of 608 DEPs), response to novelty (false discovery rate, FDR = 3.5 × 10⁻⁵), learning and memory (FDR = 8.9 × 10⁻⁷), social investigation (FDR = 0.0235), and anxiety (FDR = 0.0077) (Fig. 2C). GOChord analysis highlighted DEPs enriched across two or more phenotypes, including phosphatase and tensin homolog (PTEN; *Pten*), brain-derived neurotrophic factor receptor (TrkB; *Ntrk2*), and dynamin-1 (DNM1; *Dnm1*) (Fig. 2D).

Gene Ontology (GO) biological process (BP) analysis showed that DEPs were enriched in ASD-relevant processes, including synaptic signaling (FDR = 2.79 × 10⁻⁶), nervous system development, and regulation of dendritic spine development (Fig. 2E). Cellular component (CC) analysis further indicated predominant localization of DEPs to neuronal compartments, including synapses (FDR = 1.35 × 10⁻²⁸), axons, and dendrites (Fig. 2F).

Pathway analysis identified significant enrichment of neuronal pathways, including the glutamatergic synapse (FDR = 0.00019) and the γ-aminobutyric acid (GABA)ergic synapse (FDR = 2.64 × 10⁻⁵) (Fig. 2G). Metabolic pathways were also enriched, including aminoacyl-tRNA biosynthesis, oxidative phosphorylation, and amino acid metabolism, as well as additional pathways such as circadian entrainment, mRNA processing, and thermogenesis (Fig. 2H). Proteins associated with neuronal pathways were predominantly downregulated, whereas those associated with metabolic pathways were mainly upregulated.

Full lists of DEPs and enriched terms in *Cntnap2* mice are provided in Supplementary File 1.

### Proteomics analysis of differentially expressed proteins in the cortex of *Shank3 * mice

**Fig. 3:**
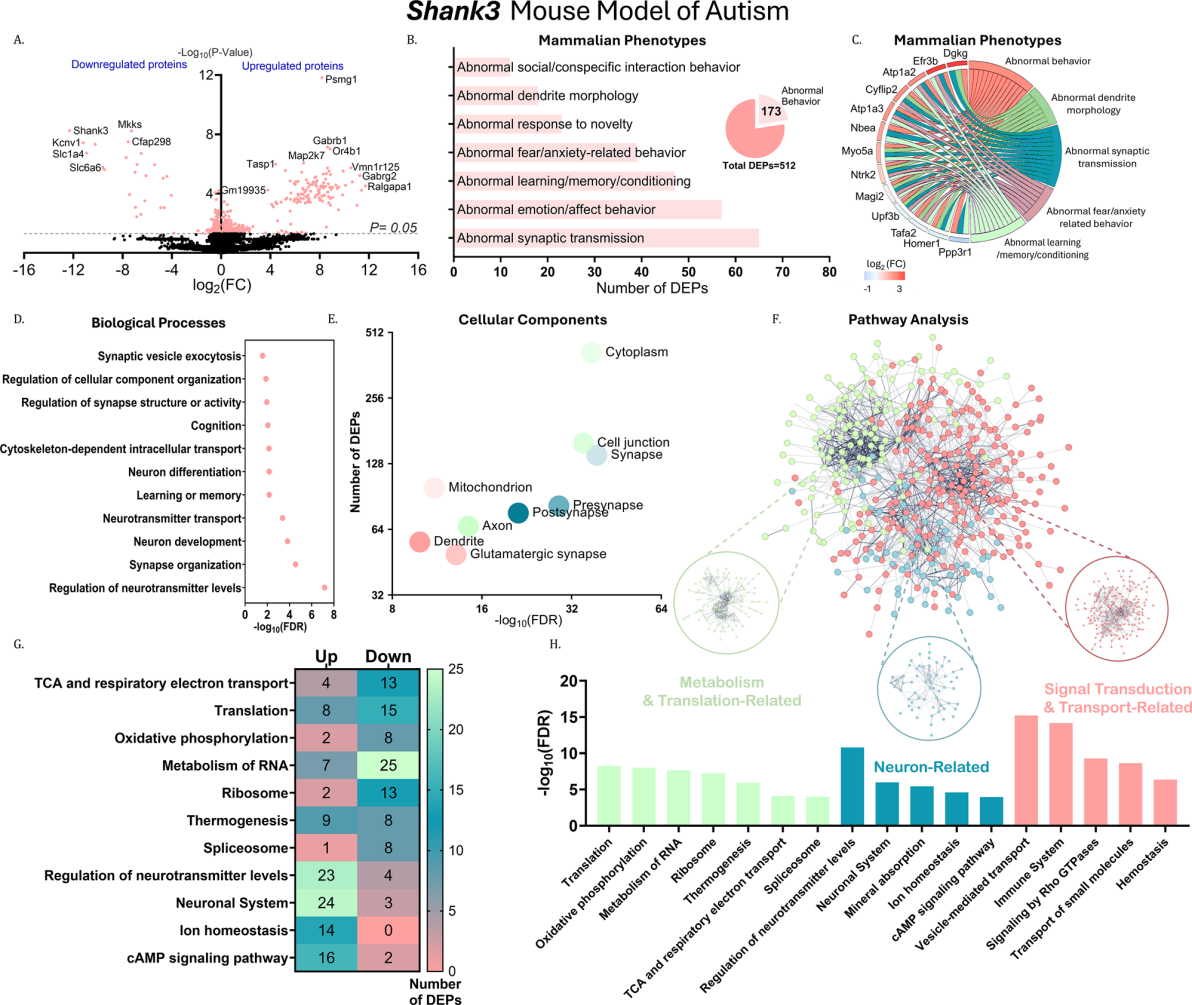
Quantitative analysis of DEPs in the cortex of *Shank3* mice. (**A**) Volcano plot of differentially expressed proteins (DEPs) in the cortex of *Shank3*/*Shank3*-WT littermates. The x-axis shows log₂(fold change, FC) with upregulated proteins on the right and downregulated proteins on the left. The y-axis shows − log₁₀(P-value). The horizontal dashed line indicates the significance threshold (P = 0.05). (B–H) Gene Ontology (GO) analysis of DEPs identified in *Shank3* mice. (**B**) Bar graph for mammalian phenotypes, with the scale representing the number of DEPs. The pie chart shows the number of DEPs associated with abnormal behavior. (**C**) GOChord diagram for mammalian phenotypes, (color scale represents log₂(FC)). (**D**) Dot plot for biological processes, with the scale representing − log₁₀(false discovery rate, FDR). (**E**) Bubble plot for cellular components, with the x-axis representing − log₁₀(FDR) and the y-axis representing the number of DEPs. (**F**) k-means clustering analysis of DEPs in the cortex of *Shank3* mice with k = 3. Protein–protein interaction enrichment shows a P-value < 1.0 × 10⁻¹⁶, with 1580 observed edges, 968 expected edges, an average node degree of 7.75, an average local clustering coefficient of 0.413, and 408 nodes. (**G**) Heatmap of clustered DEPs. (**H**) Bar graph for enriched pathways, with the scale representing  − log₁₀(FDR)

In the cortex of *Shank3* mice, 8,754 proteins were detected, of which 512 were DEPs, visualized by volcano plot analysis (Fig. 3A). Mammalian phenotype ontology analysis revealed strong enrichment of ASD-associated behavioral phenotypes, including abnormal behavior (173 of 512 DEPs; FDR = 1.13 × 10⁻¹⁰), abnormal fear/anxiety-related behavior (39 DEPs), abnormal response to novelty (23 DEPs), and abnormal social/conspecific interaction behavior (12 DEPs) (Fig. 3B). GOChord analysis highlighted DEPs enriched across multiple ASD-related behavioral phenotypes, including neurobeachin (NBEA; *Nbea*) and TrkB (Fig. 3C).

GO: BP analysis identified enrichment of neuronal processes, including neuron differentiation (FDR = 0.0068), neurotransmitter transport (FDR = 4 × 10⁻⁴), and synapse organization (FDR = 2.89 × 10⁻⁵) (Fig. 3D). CC analysis further indicated predominant localization of DEPs to neuronal compartments, with enrichment in synapses (27% of DEPs), axons (13%), and dendrites (11%) (Fig. 3E).

K-means clustering segregated DEPs into three distinct clusters with divergent functional clusters (Fig. 3F). The neuron-related cluster was enriched for pathways involved in regulation of neurotransmitter levels (FDR = 1.54 × 10⁻¹¹), ion homeostasis, and cAMP signaling. A second cluster was enriched for metabolic and translational pathways, including the citric acid cycle and respiratory electron transport (FDR = 7.41 × 10⁻⁵) and oxidative phosphorylation. The third cluster was enriched for signal transduction and transport pathways, including immune system–related processes (FDR = 6.7 × 10⁻¹⁵) and post-translational protein modification (Figs. 3F–H). The distribution of up- and downregulated DEPs across these pathways is shown in Fig. 3G. Full lists of DEPs and enriched terms in *Shank3* mice are provided in Supplementary File 2.

### Comparative analysis of DEPs and gene ontology in *Cntnap2* and *Shank3* autism mouse models

**Fig. 4:**
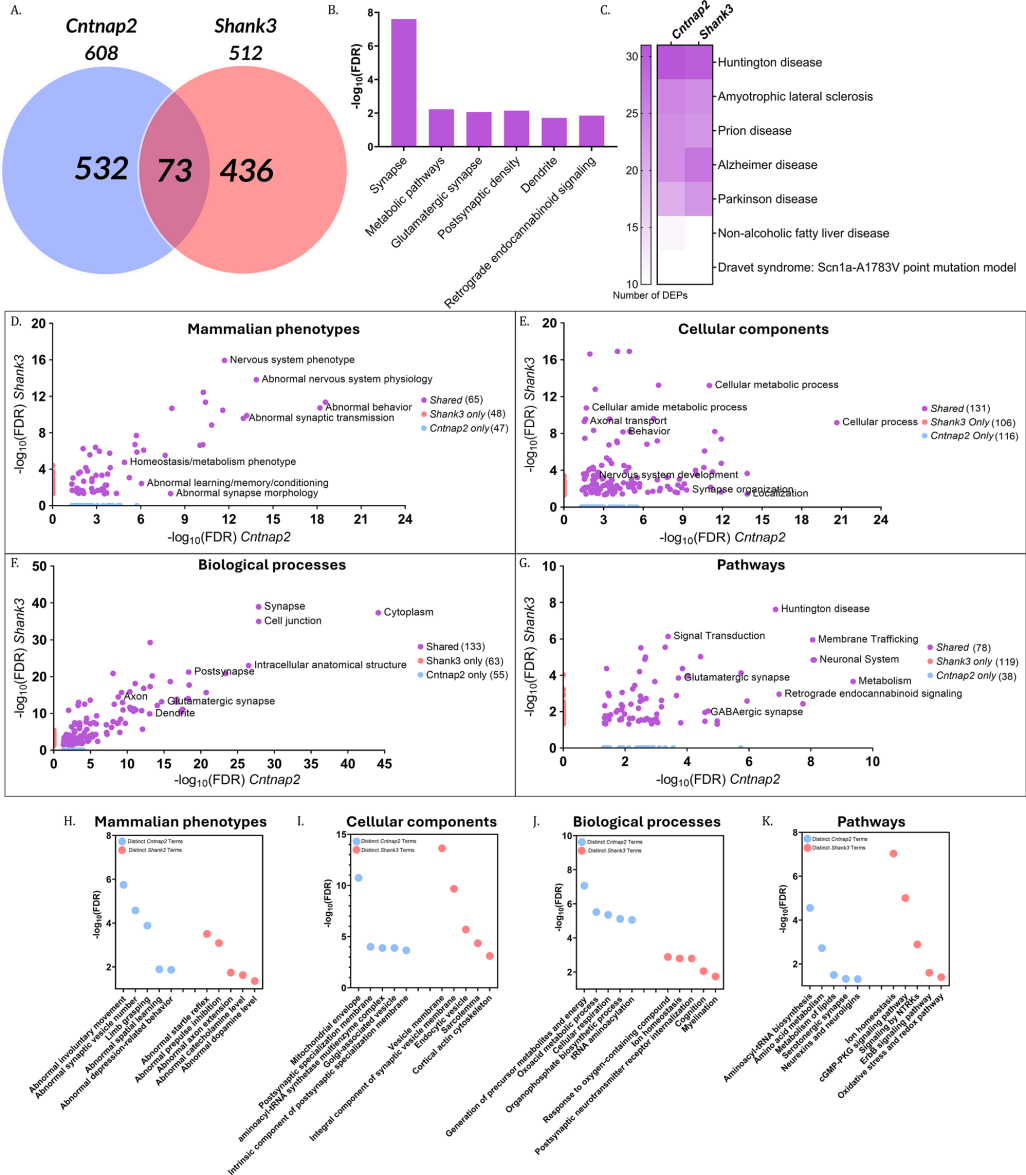
Comparative analysis of DEPs and Gene Ontology in *Cntnap2* and *Shank3* ASD mouse models (**A**). Venn diagram illustrating the number of differentially expressed proteins (DEPs) in the *Cntnap2* and *Shank3* mouse models compared with their WT littermates. Blue and red circles represent DEPs identified in the cortex of *Cntnap2* and *Shank3* mice, respectively. (**B**) Gene Ontology (GO) analysis of DEPs shared between *Cntnap2* and *Shank3* models, with the scale representing −log₁₀(false discovery rate, FDR). (**C**) Heatmap showing the number of DEPs enriching disease-related terms in *Cntnap2* and *Shank3* mice, with the scale representing the number of DEPs. (**D**–**G**) Scatter plots showing enriched terms in *Cntnap2* only (blue), *Shank3* only (red), or shared between both models (purple) for (**D**) mammalian phenotypes, (**E**) cellular components, (**F**) biological processes, and (**G**) pathways. The x-axis represents −log₁₀(FDR) in *Cntnap2* mice and the y-axis represents −log₁₀(FDR) in *Shank3* mice. (H–K) Scatter plots showing distinct enriched terms in *Cntnap2* (blue) and *Shank3* (red) mice for (**H**) mammalian phenotypes, (**I**) biological processes, (**J**) cellular components, and (**K**) pathways. The scale represents −log₁₀(FDR)

Comparative analysis of the two ASD models identified 73 shared DEPs between *Cntnap2* and *Shank3* mice, corresponding to 14.26% of DEPs in the *Shank3* cortex (Jaccard overlap index = 6.98%) (Fig. 4A). GO analysis of these shared DEPs revealed enrichment of synaptic and metabolic terms, including synapse, glutamatergic synapse, and metabolic pathways (Fig. 4B).

Disease association analysis further showed that a subset of shared DEPs mapped to multiple neurodegenerative disease terms, including Alzheimer’s and Parkinson’s disease (Fig. 4C). These DEPs clustered predominantly within mitochondrial complexes I, III, and IV, corresponding to oxidative phosphorylation. The full list of proteins is provided in Supplementary File 3.

Comparative scatter plot analyses across mammalian phenotypes, CC, BP, and pathways demonstrated extensive overlap between the two models (Figs. 4D–G). In total, 65 phenotypes, 133 CCs, 131 BPs, and 78 pathways were shared. Common phenotypes included abnormal behavior, learning/memory deficits, and abnormal emotion/affect behavior. Shared CCs encompassed cytoplasm, endosomes, dendritic spines, and synapses, while shared BPs involved neuronal development, differentiation, and synapse formation. Shared pathways included GABAergic and glutamatergic signaling, metabolic processes, and mRNA processing.

In addition to shared features, each model displayed distinct signatures. *Cntnap2* mice showed unique enrichments associated with motor phenotypes, aminoacyl-tRNA-related cellular components, and serotonergic synapse pathways (Figs. 4H–K). In contrast, *Shank3* mice exhibited unique enrichments related to sensorimotor and startle phenotypes, ion homeostasis-related BPs, and oxidative stress-associated pathways (Figs. 4H–K).

Detailed lists of shared and model-specific DEPs and enriched terms are provided in Supplementary File 3.

### Effects of 7-NI on the cortical protein expression landscape of *Cntnap2* and *Shank3* mutant mice

**Fig. 5:**
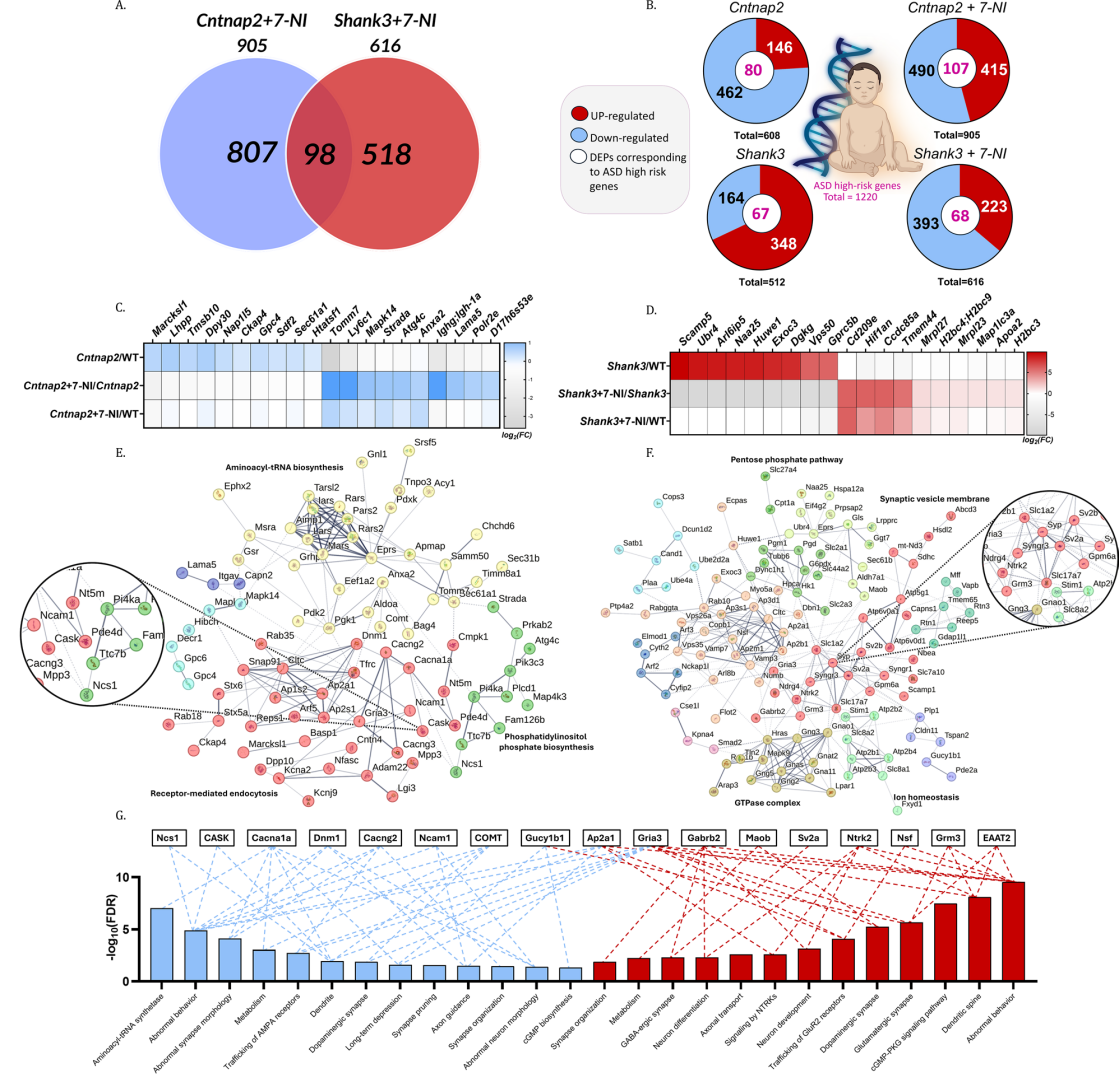
Comparative analyses of DEPs in the cortex of *Cntnap2* and *Shank3* mice treated with 7-NI (**A**) Venn diagram illustrating the number of differentially expressed proteins (DEPs) in the *Cntnap2* and *Shank3* mouse models of autism spectrum disorder treated with 7-Nitroindazole (7-NI), compared with untreated mutants. Blue and red circles represent DEPs identified in the cortex of *Cntnap2*+7-NI and *Shank3*+7-NI mice, respectively. (**B**) Pie charts show the number of upregulated and downregulated proteins in the cortex of *Cntnap2* and *Shank3* mice compared with their WT counterparts, as well as *Cntnap2*+7-NI and *Shank3*+7-NI mice compared with untreated mutants. Numbers within the white circles indicate the count of DEPs corresponding to high-confidence autism spectrum disorder risk genes. (**C**–**D**) Heatmaps showing log₂(fold change) of DEPs in the cortex of (**C**) the *Cntnap2* cohort (*Cntnap2* /WT, *Cntnap2*+7-NI*/Cntnap2*, and *Cntnap2*+7-NI/WT) and (**D**) the *Shank3* cohort (*Shank3*/WT, *Shank3*+7-NI/*Shank3*, and *Shank3*+7-NI/WT). (**E**–**F**) Markov Cluster Algorithm (MCL) clustering analysis of partially rescued DEPs in the cortex of (**E**) *Cntnap2*+7-NI mice with an inflation parameter of 1.3 and (**F**) *Shank3*+7-NI mice with an inflation parameter of 1.4. Protein–protein interaction enrichment shows a P-value < 1.0 × 10⁻¹⁶. Observed and expected edges, average node degree, average local clustering coefficient, and the number of nodes are indicated. (**G**) Gene Ontology (GO) analysis showing biological processes, pathways, and phenotypes enriched by partially rescued DEPs in *Cntnap2* mice shown as blue bars and *Shank3* mice shown as red bars. Key proteins are listed and linked to the GO terms they enrich. The y-axis represents −log₁₀(false discovery rate, FDR).

Mutant mice were treated with 7-NI, a selective nNOS inhibitor [14, 46], and proteomic analysis identified 905 DEPs in *Cntnap2* + 7-NI mice and 616 DEPs in *Shank3* + 7-NI mice relative to their respective untreated mutants (Fig. 5A). Comparative analysis identified 98 DEPs commonly altered by 7-NI in both models (Jaccard overlap index = 6.89%). These shared DEPs were enriched for behavioral, neuronal, and synaptic terms (Supplementary File. 4).

Proteomic datasets from untreated and 7-NI–treated mutants were compared with high-risk ASD genes from the Simons Foundation Autism Research Initiative (SFARI) database. A significant overlap was observed between DEPs and ASD-risk genes, with 80 DEPs in *Cntnap2* mice, 67 in *Shank3* mice, 107 in *Cntnap2*+7-NI mice, and 68 in *Shank3*+7-NI mice encoded by high-risk ASD genes (Fig. 5B). Representative genes include PTEN [50], *CDH10* [51], *SCN2A* [52], *MECP2* [53], and *SYNCRIP* [54]. Comparison with human orthologs using BioMart indicated high sequence conservation (> 90% on average) (Supplementary File 4).

To assess proteomic shifts toward WT levels, we examined DEPs that were no longer significantly different from WT with 7-NI treatment (Figs. 5C–F). In *Cntnap2* mutants, 367 of 608 DEPs, and in *Shank3* mutants, 445 of 512 DEPs, were no longer significant following 7-NI treatment, while additional DEPs emerged uniquely in the treated groups. Heatmaps illustrate representative proteins shifted towards WT levels in *Cntnap2*+7-NI and *Shank3*+7-NI mice (Figs. 5C, D). In *Cntnap2* mice, examples included the mitochondrial import receptor subunit (TOMM7; *Tomm7*), MARCKS-related protein (MARCKSL1; *Marcksl1*), neuronal calcium sensor 1 (NCS1; *Ncs1*), and leucine-rich repeat LGI family member 3 (LGI3; *Lgi3*). In *Shank3* mice, proteins showing similar shifts included secretory carrier-associated membrane protein 5 (SCAMP5; *Scamp5*), ubiquitin-protein ligase (UBR4; *Ubr4*), and monoamine oxidase B (MAO-B; *Maob*). Functional clustering of rescued proteins identified enrichment of phosphatidylinositol phosphate biosynthesis, receptor-mediated endocytosis, and aminoacyl-tRNA biosynthesis in *Cntnap2* mice (Fig. 5E), and synaptic vesicle membrane, pentose phosphate pathway, ion homeostasis, and GTPase complex in *Shank3* mice (Fig. 5F).

An association network linked partially rescued proteins to enriched behavioral, developmental, and synaptic categories in each ASD model (Fig. 5G).

Full lists of DEPs, enriched terms, and partially rescued proteins are provided in Supplementary Files 1, 2, and 4.

### Gene ontology analysis of the partially rescued DEPs in ASD-related phenotypes

**Fig. 6:**
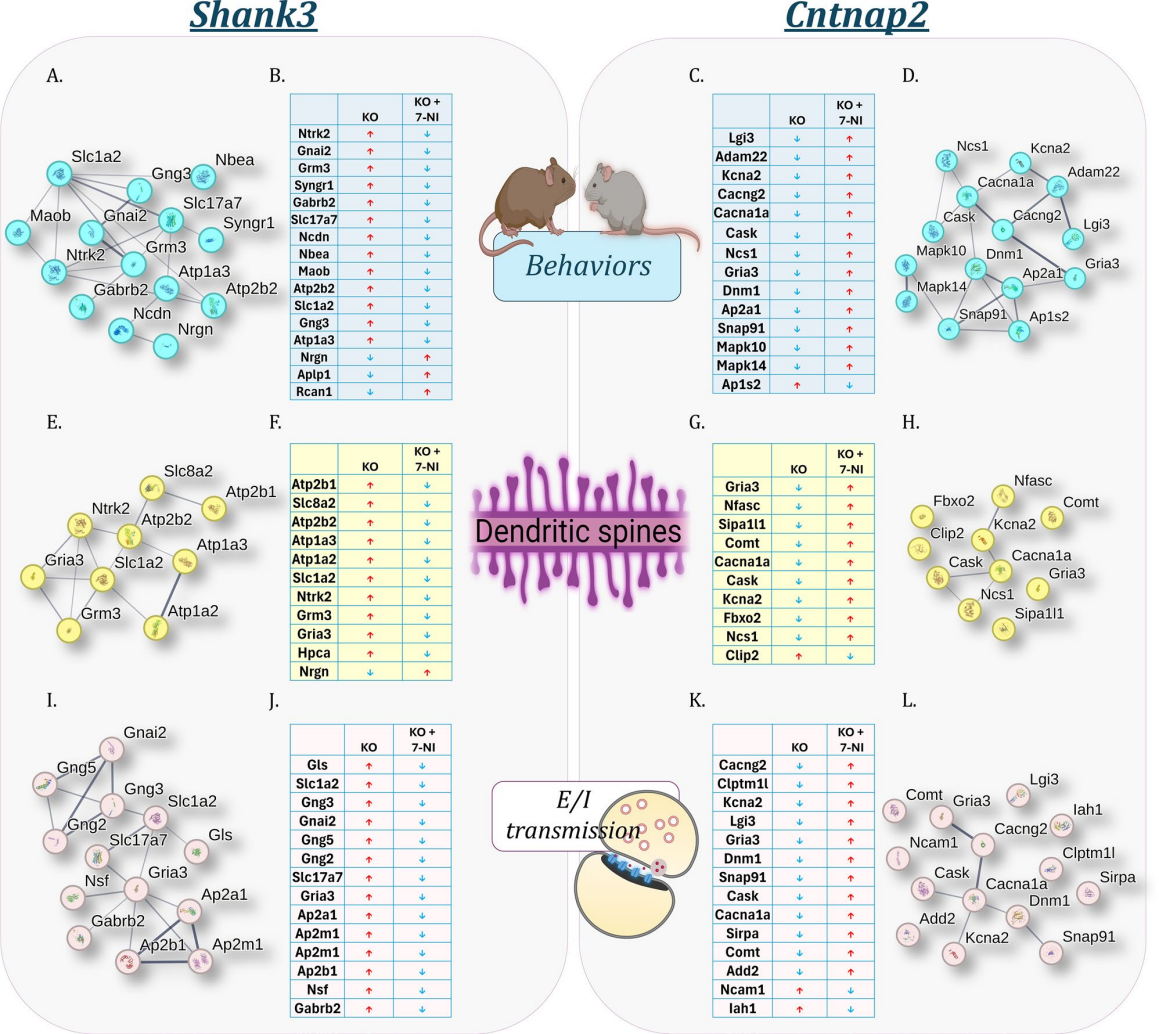
Effects of 7-NI on behavior, spine density, and synaptic transmission in *Shank3* and *Cntnap2* mice. Clustering analyses and tables showing differentially expressed proteins (DEPs) related to behavior that were partially rescued by 7-Nitroindazole (7-NI) treatment. (**A**–**B**) Behavioral-related DEPs in *Shank3* mice with protein–protein interaction (PPI) enrichment P < 1.0 × 10⁻¹⁶, 20 observed edges, 1 expected edge, an average node degree of 2.86, an average local clustering coefficient of 0.477, and 14 nodes. (**C**–**D**) Behavioral-related DEPs in *Cntnap2* mice with PPI enrichment P = 5.6 × 10⁻¹³, 19 observed edges, 2 expected edges, an average node degree of 2.71, an average local clustering coefficient of 0.471, and 14 nodes. (**E**–**F**) Dendritic spine–related DEPs in *Shank3* mice with PPI enrichment P < 1.0 × 10⁻¹⁶, 13 observed edges, 0 expected edges, an average node degree of 2.89, an average local clustering coefficient of 0.47, and 9 nodes. (**G**–**H**) Dendritic spine-related DEPs in *Cntnap2* mice with PPI enrichment P = 3.72 × 10⁻⁵, 5 observed edges, 0 expected edges, an average node degree of 1.0, an average local clustering coefficient of 0.333, and 10 nodes. (**I**–**J**) Excitation-inhibition (E/I) transmission–related DEPs in *Shank3* mice with PPI enrichment P = 2.09 × 10⁻¹³, 19 observed edges, 2 expected edges, an average node degree of 2.92, an average local clustering coefficient of 0.835, and 13 nodes. (**K**–**L**) E/I transmission–related DEPs in *Cntnap2* mice with PPI enrichment P = 0.0016, 6 observed edges, 1 expected edge, an average node degree of 0.857, an average local clustering coefficient of 0.286, and 14 nodes. *Cntnap2* and *Shank3* mice were compared with their WT littermates, while *Cntnap2*+7-NI and *Shank3*+7-NI mice were compared with their respective untreated mutant controls.  Up-regulated protein  Down-regulated protein

Three functional domains (behavior, dendritic spine regulation, and excitation–inhibition (E/I) neurotransmission) were examined to identify DEPs shifting toward WT levels following 7-NI treatment (Fig. 6).

Within the behavioral domain, proteins shifting toward WT levels in *Shank3* + 7-NI mice included metabotropic glutamate receptor 3 (mGluR3; *Grm3*), the γ-aminobutyric acid (GABA) type A receptor β2 subunit (GABRB2; *Gabrb2*), excitatory amino acid transporter 2 (EAAT2; *Slc1a2*), and TrkB (Figs. 6A and B). In *Cntnap2* + 7-NI mice, examples included potassium voltage-gated channel (Kv1.2; *Kcna2*), glutamate receptor 3 (GluA3; *Gria3*), DNM1 and LGI3 (Figs. 6C and D).

In the dendritic spine regulation domain, proteins shifting toward WT levels in *Shank3* + 7-NI mice included TrkB, GluA3, mGluR3, and neurogranin (NRGN; *Nrgn*) (Figs. 6E & 6F). Corresponding proteins in *Cntnap2* + 7-NI mice included neurofascin (NFASC; *Nfasc*), catechol-O-methyltransferase (COMT; *Comt*), the P/Q-type voltage-gated calcium channel (CaV2.1; *Cacna1a*), Calcium/calmodulin-dependent serine protein kinase (CASK; *Cask*), and GluA3 (Figs. 6G & 6H).

Within the E/I neurotransmission domain, *Shank3* + 7-NI mice showed shifts toward WT levels for multiple synaptic vesicle- and neurotransmission-related proteins, including synaptic vesicle glycoproteins (SV2A; *Sv2a*), synaptophysin (SYP; *Syp*), glutaminase (GLS; *Gls*), vesicular glutamate transporter 1 (VGLUT1; *Slc17a7*), and GABRB2 (*Gabrb2*) (Figs. 6I and J). In *Cntnap2* + 7-NI mice, proteins showing similar shifts included calcium channel γ2 subunit (CaVγ2; *Cacng2*), LGI3, GluA3, DNM1, and CASK (Figs. 6K and L).

Together, these analyses show domain-specific proteomic shifts toward WT levels in *Cntnap2* and *Shank3* mice with 7-NI treatment.

### Orthogonal western blot validation and comparative analysis with proteomics data

**Fig. 7:**
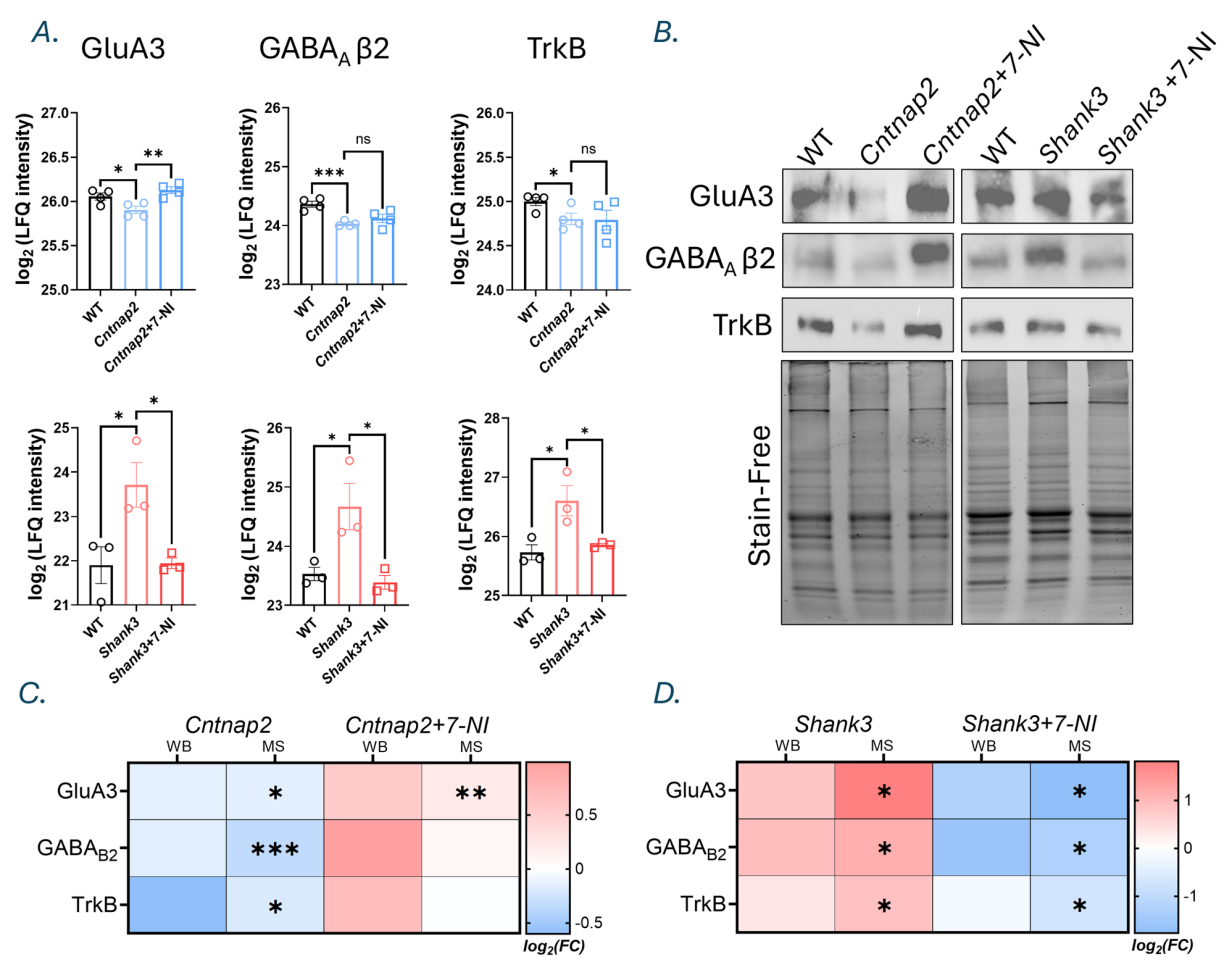
Western blot measurements for selected proteins and Comparison with mass spectrometry. (**A**) Bar graphs showing log₂-transformed label-free quantification (LFQ) intensities of GluA3, GABAA receptor β2 subunit (GABRB2), and full-length TrkB (TrkB-FL) measured by mass spectrometry. Data are shown for WT, *Cntnap2*, and *Cntnap2*+7-Nitroindazole (7-NI) mice (left, n = 3), and for *Shank3* WT littermate controls, *Shank3*, and *Shank3*+7-NI mice (right, n = 4). Dots represent individual animals, and bars indicate mean ± SEM. Statistical analysis was performed using unpaired two-tailed t-tests for predefined comparisons (WT vs mutant and mutant vs mutant+7-NI). Uncorrected P-values are shown. (**B**) Representative Western blots of GluA3, GABRB2, and TrkB-FL. Signal intensities were normalized to total protein per lane using stain-free imaging of the same membrane. Lanes are shown as WT, *Cntnap2*, and *Cntnap2*+7-NI (left, n = 3), and *Shank3* WT littermate, *Shank3*, and *Shank3*+7-NI (right, n = 3). Western blotting was used as an orthogonal approach to support the proteomic findings. (**C**–**D**) Heatmaps comparing log₂ fold changes measured by Western blotting and mass spectrometry for the *Cntnap2* (**C**) and *Shank3* (**D**) groups. Western blot quantification was analyzed using one-way ANOVA followed by Dunnett’s multiple-comparisons test. ns, not significant; *, p < 0.05; **, p < 0.01; ***, p < 0.001

Three proteins, GluA3, GABRB2, and TrkB were selected for orthogonal validation. Label-free quantification (LFQ) intensities derived from MS–based proteomic analysis are shown as bar graphs for WT, knock-out (KO), and KO + 7-NI in both the *Cntnap2* and *Shank3* models (Fig. 7A). Western blot (WB) analyses were performed on cortical lysates from the same experimental groups, and protein levels were quantified for each target (Fig. 7B). Comparison of log_2_(fold changes) obtained from WB and MS revealed directionally consistent changes across methods (Figs. 7C and D); however, the WB differences did not reach statistical significance. All uncropped Western blots and band quantifications are provided in Supplementary File 5.

## Discussion

*Cntnap2* and *Shank3* are two autism spectrum disorder (ASD) mouse models exhibiting dysregulated nitric oxide (NO) signaling. Pharmacological inhibition of neuronal nitric oxide synthase (nNOS) by 7-Nitroindazole (7-NI) is associated with improvements in the ASD-like phenotypes in both models (Fig. 1). However, the molecular mechanisms underlying this effect remain incompletely understood. Therefore, in this work, we applied global proteomics and systems biology analyses to investigate the molecular changes associated with the ASD-like behaviors in these models.

Our analyses revealed enrichments of ASD-related behavioral phenotypes in both mutant models, including sociability-related behaviors, anxiety, and learning and memory. This is consistent with behavioral dysfunction being the defining feature of ASD [55–60]. NO is a neuromodulator implicated in the regulation of these behaviors; moreover, excessive NO in the brain has been associated with anxiety-like behaviors, depression, and cognitive impairment [60, 61]. Furthermore, our previous work showed that pharmacological elevation of NO levels in wild-type (WT) mice induced ASD-like behavioral deficits comparable to those observed in *Cntnap2* and *Shank3* mouse models [14]. Several differentially expressed proteins (DEPs) contributing to the behavioral enrichments showed partial shifts toward WT levels following nNOS inhibition with 7-NI in both models. For example, TrkB, a receptor tyrosine kinase for neurotrophins [62] which has been associated with ASD traits [63], exhibited partial normalization toward WT levels in *Shank3* mutants. NO has been reported to modulate TrkB receptor function through nitration of a specific tyrosine residue [64]. Moreover, dysregulation of TrkB signaling has been linked to psychiatric disorders [65, 66]. Another example is postsynaptic protein NRGN [67]. NO has been shown to modify NRGN, potentially through S-Nitrosylation (SNO), which can interact with its phosphorylation and alter its structure and functional properties [68]. Notably, *NRGN* KO mice exhibit behavioral deficits that are characteristic of some neuropsychiatric disorders, including ASD [69]. Together, these findings suggest that nNOS inhibition may influence ASD-relevant behaviors, potentially through modulation of multiple synaptic proteins.

At the neurodevelopmental level, our analyses revealed enrichment of neurodevelopmental processes in both *Cntnap2* and *Shank3*, including neuronal development, morphology, differentiation, and neurogenesis. These findings align with prior reports showing that the two mutants exhibit developmental impairments, including neuronal migration abnormalities in *Cntnap2* and reduced brain volume and white matter alterations in *Shank3* [70–72]. This is noteworthy, as ASD-related behaviors are thought to arise from disruptions in neurodevelopmental processes [73–77]. While physiological NO is necessary for normal cortical development, excessive nitrosative stress has been shown to disrupt DNA binding of essential transcription factors, interfering with neurogenesis, differentiation, and maturation and the proper migration of neurons [78]. Several DEPs underlying these enrichments were shifted towards WT levels in 7-NI-treated mutant models. These DEPs include NCS1, a calcium sensor that regulates neurogenesis and neuronal morphology [79], neural cell adhesion molecule 1 (NCAM1, *Ncam1* gene), a cell surface adhesion glycoprotein that is crucial for neuron connectivity, neuron migration, and axonal growth [80, 81].

At the synaptic level, a substantial proportion of the altered cortical proteins identified in both *Cntnap2* and *Shank3* mice localized to synaptic compartments and were enriched for processes related to both glutamatergic and γ-aminobutyric acid (GABA)ergic synapses, in addition to synapse morphology, assembly, and organization (Figs. 2E and 3D). Some of these features were previously observed in these mice [14–16, 34]. Synaptic alterations in ASD are often associated with excitation-inhibition (E/I) imbalance arising from disrupted glutamatergic and GABAergic neurotransmission [82, 83]. In line with this, and consistent with the evidence that excessive NO signaling may exert deleterious effects on synaptic transmission [84], our analyses revealed normalization of several related proteins following nNOS inhibition. Examples include the α-amino-3-hydroxy-5-methyl-4-isoxazolepropionic acid (AMPA) receptor subunit GluA3, and the GABA_A_ receptor β2 subunit (GABRB2). Alterations in GluA3 have been shown to affect synaptic plasticity and excitatory neurotransmission and, when persistent, can be associated with neuronal morphological changes and cognitive impairments [85–87]. Likewise, alterations in GABRB2 disrupt inhibitory neurotransmission and have been linked to neurodevelopmental disorders [88–90], with both proteins implicated in ASD-related phenotypes [91–95]. Together, these findings suggest that dysregulated NO signaling may influence the molecular composition of excitatory and inhibitory synapses in ASD. Beyond synaptic receptors, nNOS inhibition was associated with partial shifts toward WT levels in proteins with central roles in neurotransmitter metabolism, transport, and clearance in both models, many of which are targets of approved neuropsychiatric medications [96–99]. These include MAO-B [100], postsynaptic metabolizer COMT [101], the norepinephrine transporter NET [97], and astrocytic transporters EAAT1 and EAAT2 [102]. Together, these findings suggest that nNOS inhibition may influence multiple neurotransmitter-regulatory mechanisms relevant to behavioral and cognitive dysfunction.

At the structural level, ASD has been associated with decreased density of mature dendritic spines [103], small protrusions extending from dendrites that are crucial for synaptic communication and plasticity. Our work revealed enrichment in dendritic spine regulation as well as alterations in dendritic spine-related proteins in both ASD models. Previous experiments showed reduced dendritic spine numbers in cortical neurons of *Cntnap2* and *Shank3* mice, which were restored by 7-NI treatment [14]. Accordingly, NO promotes spine formation, growth, and stability, but can also contribute to spine loss and degeneration through complex signaling pathways [104–106]. Some of the alterations in dendritic spine-related proteins in the two ASD models were rescued by 7-NI. Representative examples include CaV2.1, a voltage-dependent calcium channel encoded by the high-risk ASD gene *CACNA1A* [107]. NO has been reported to bidirectionally modulate CaV2.1 activity, producing either inhibition or potentiation depending on cellular context, likely via cGMP/PKG-dependent signaling [108]. *CACNA1A*^*+/−*^ neurons exhibited decreased synaptic density [109]. Another is CASK, which is implicated in dendritic spine organization and stability [110]. *Cask* gene overexpression stabilized and maintained spine morphology [110]. These molecular changes are consistent with the previously reported restoration of dendritic spine density in mutant cortices following nNOS inhibition.

At the metabolic level, both *Cntnap2* and *Shank3* ASD mouse models exhibit enrichment of metabolic pathways (Figs. 2G and 3H), consistent with previous reports [111–113]. Notably, metabolic alterations and dysfunction are increasingly recognized in ASD, affecting up to 30% of ASD children [114], such as altered triglycerides, low-density lipoprotein (LDL) cholesterol, and elevated lactate or lactate-to-pyruvate ratios [115, 116]. A molecular example is lipid signaling, which regulates synaptic transmission, plasticity, and neurodevelopment through second-messenger pathways, and its disruption can impair synaptic function and neuronal stability [117]. In this context, nitrosative stress has been shown to disrupt cellular metabolism by impairing mitochondrial function and altering glucose and lipid homeostasis [118]. Importantly, the rescue effects of nNOS inhibition extended beyond neuronal proteins to include metabolic ones. An example is phosphatidylinositol 4-kinase-α (PI4Kα), a brain-expressed lipid kinase essential for brain development and myelination [119, 120]. PI4Kα was shown to be impacted by NO through modulation of phosphoinositide signaling [121, 122]. PI4Kα loss causes severe neurodevelopmental abnormalities, including developmental delay, and intellectual disability [120]. These findings suggest a potential link between NO-dependent metabolic regulation and ASD pathology in *Cntnap2* and *Shank3* mutants.

NO signaling mediates many of its effects through intracellular second-messenger pathways [123], prompting us to examine downstream NO-associated cascades. One such pathway is cGMP–PKG signaling, a canonical effector of NO [124]. Previous work from our group showed elevated cortical cGMP levels in both models, which were normalized following nNOS inhibition [14]. Consistent with this, our proteomic analyses indicate alterations in components of the cGMP-PKG pathway in the mutant models, several of which showed normalization following nNOS inhibition, supporting modulation of downstream NO signaling.

To assess translational relevance, DEPs identified in both mouse models were compared with high-confidence ASD genes from the Simons Foundation Autism Research Initiative (SFARI) database, revealing significant overlap. Notably, most of the affected proteins are highly conserved between mice and humans. One example is NCAM1, which showed partial normalization following treatment, NCAM1 exhibits high evolutionary conservation, consistent expression across cortical regions implicated in ASD, and dynamic regulation during neurodevelopment. In addition, our previous analyses of human ASD samples demonstrated elevated plasma levels of 3-nitrotyrosine, a marker of nitrosative stress, along with altered SNO profiles [14]; however, plasma and brain represent distinct biological compartments. Collectively, these findings suggest that nNOS inhibition by 7-NI may modulate molecular pathways relevant to human ASD.

In conclusion, proteomic analysis of two genetically distinct ASD mouse models reveals convergent disruption of NO-sensitive neuronal, synaptic, and metabolic pathways in the cortex, which partially shift toward wild-type states following nNOS inhibition. These findings highlight partially convergent downstream mechanisms despite different primary mutations and provide a framework for future genetic and translational studies to define causal roles of NO signaling in ASD.

### Limitations

This study provides molecular insight into ASD-related alterations in *Cntnap2* and *Shank3* mouse models and the role of nitric oxide (NO) signaling. Several limitations should be considered.

Mass spectrometry enables broad proteome coverage but may have reduced sensitivity for low-abundance proteins [125]. In addition, our analyses focused on the cortex, a key region in ASD [126]; however, extending profiling to other brain regions such as the striatum [127], amygdala [128], and hippocampus [129, 130] may offer additional insights [131, 132].

Although ASD is genetically heterogeneous and no single model fully captures the disorder [133], the *Cntnap2* and *Shank3* models reproduce core ASD-related features [134, 135]. Increasing sample size and incorporating additional models may further strengthen future studies.

Finally, while 7-Nitroindazole (7-NI) is a well-investigated and relatively selective nNOS inhibitor, it may exhibit off-target effects, particularly at higher doses. Therefore, future studies would benefit from assessing its selectivity both in vitro and in vivo, exploring alternative more selective nNOS inhibitors if available, or employing genetic models such as nNOS knockout (KO) mice.

## Conclusions

Our previous work linked nitric oxide (NO) dysregulation to ASD-like phenotypes in *Cntnap2* and *Shank3* mouse models, which were partially reversed by nNOS inhibition. In this study, high-throughput proteomic and bioinformatic analyses revealed widespread protein expression changes in cortical pathways related to synaptic transmission, development, and metabolism in both models. nNOS inhibition with 7-Nitroindazole partially normalized many of these alterations, including proteins involved in GABAergic and glutamatergic signaling. Notably, overlapping differentially expressed proteins and pathways were observed across both models, indicating convergent molecular mechanisms. The enrichment of ASD-risk gene-encoded proteins among nNOS-responsive targets suggests potential translational relevance. Together, these findings support a role for NO signaling in ASD-related molecular and behavioral phenotypes and highlight nNOS inhibition as a potential therapeutic avenue warranting further investigation.

## Supplementary Information

Below is the link to the electronic supplementary material.

Supplementary Material 1

Supplementary Material 2

Supplementary Material 3

Supplementary Material 4

Supplementary Material 5

## Data Availability

The dataset(s) supporting the conclusions of this article are included in this article and its Supplementary Files. Please see the section “Sample preparation” in the Methods for a full list of materials available and where they can be accessed. The mass spectrometry proteomics data have been deposited with the ProteomeXchange Consortium via the PRIDE repository under the dataset identifier PXD068474.

## Abbreviations

7-NI: 7–Nitroindazole
AMPA: α–Amino–3–hydroxy–5–methyl–4–isoxazolepropionic acid receptor
ANOVA: Analysis of Variance
ASD: Autism Spectrum Disorder
BCA: Bicinchoninic Acid
BDNF: Brain–Derived Neurotrophic Factor
BP: Biological Process
CC: Cellular Component
CNTNAP2: Contactin–associated protein–like 2
DEP: Differentially Expressed Protein
DMSO: Dimethyl sulfoxide
EDTA: Ethylenediaminetetraacetic Acid
E/I: Excitation/Inhibition
FC: Fold Change
FDR: False Discovery Rate
GABA: γ–Aminobutyric Acid
GO: Gene Ontology
HEPES: 4–(2–hydroxyethyl)–1–piperazineethanesulfonic Acid
IP: Intraperitoneal
KO: Knockout
LC: Liquid Chromatography
LTP: Long–Term Potentiation
MCL: Markov Cluster Algorithm
MS: Mass Spectrometry
NMDA: N–methyl–D–aspartate receptor
NO: Nitric Oxide
nNOS: Neuronal Nitric Oxide Synthase
NOS: Nitric Oxide Synthase
PMSF: Phenylmethylsulfonyl Fluoride
PVDF: Polyvinylidene Fluoride
RIPA: Radioimmunoprecipitation Assay
SDS: Sodium Dodecyl Sulfate
SDS-PAGE: Sodium Dodecyl Sulfate–Polyacrylamide Gel Electrophoresis
SFARI: Simons Foundation Autism Research Initiative
SHANK3: SH3 and Multiple Ankyrin Repeat Domains 3
SNO: S–Nitrosylation
TBST: Tris–buffered saline with Tween–20
Tris: HCl–Tris(hydroxymethyl)aminomethane Hydrochloride
WB: Western Blotting
WT: Wild–Type
