# Supplementary material for: Nitric oxide inhibition ameliorates cortical proteomic changes in the Cntnap2-/- and Shank3Δ4–22 mouse models of autism spectrum disorder
Source: Mol Autism. 2026 Apr 20;17:21. doi: 10.1186/s13229-026-00716-1 (PMC13107916; doi:10.1186/s13229-026-00716-1)
Supplement: Supplementary file 3 — Supplementary Material 3 [file 13229_2026_716_MOESM3_ESM.pdf]

Nitric Oxide Inhibition Ameliorates Cortical Proteomic Changes in the Cntnap2$^{-/-}$ and Shank3Δ4-22 Mouse Models of Autism Spectrum Disorder

Wisam Bazbaz[1], Maryam Kartawy[1], Igor Khaliulin[1], Haitham Amal[1, 2]*

1 Institute for Drug Research, School of Pharmacy, Faculty of Medicine, The Hebrew University of Jerusalem, Jerusalem, Israel.

2 Rosamund Stone Zander and Hansjoerg Wyss Translational Neuroscience Center, Boston Children's Hospital, Harvard Medical School, Boston, Massachusetts, USA.

<u>Uncropped Western Blot Images (Raw Data) & Band quantifications</u>

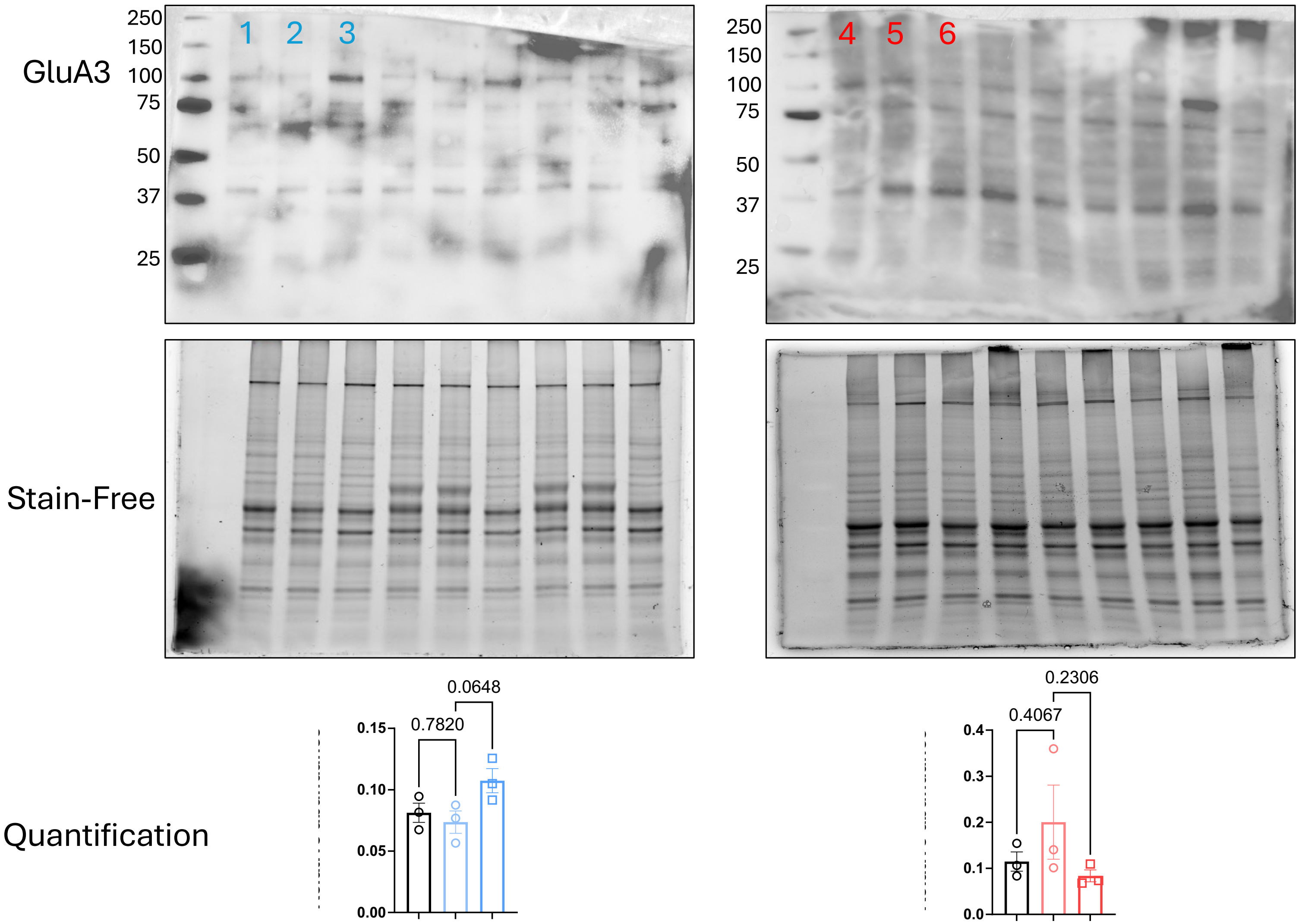

1:B6
2:*Cntnap2*
3:*Cntnap2+7-NI*
4:*Shank3* WT
5:*Shank3*
6:*Shank3*+7-NI

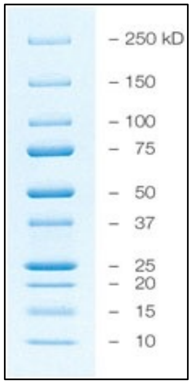

Precision Plus Protein™

All Blue Prestained Protein Standards

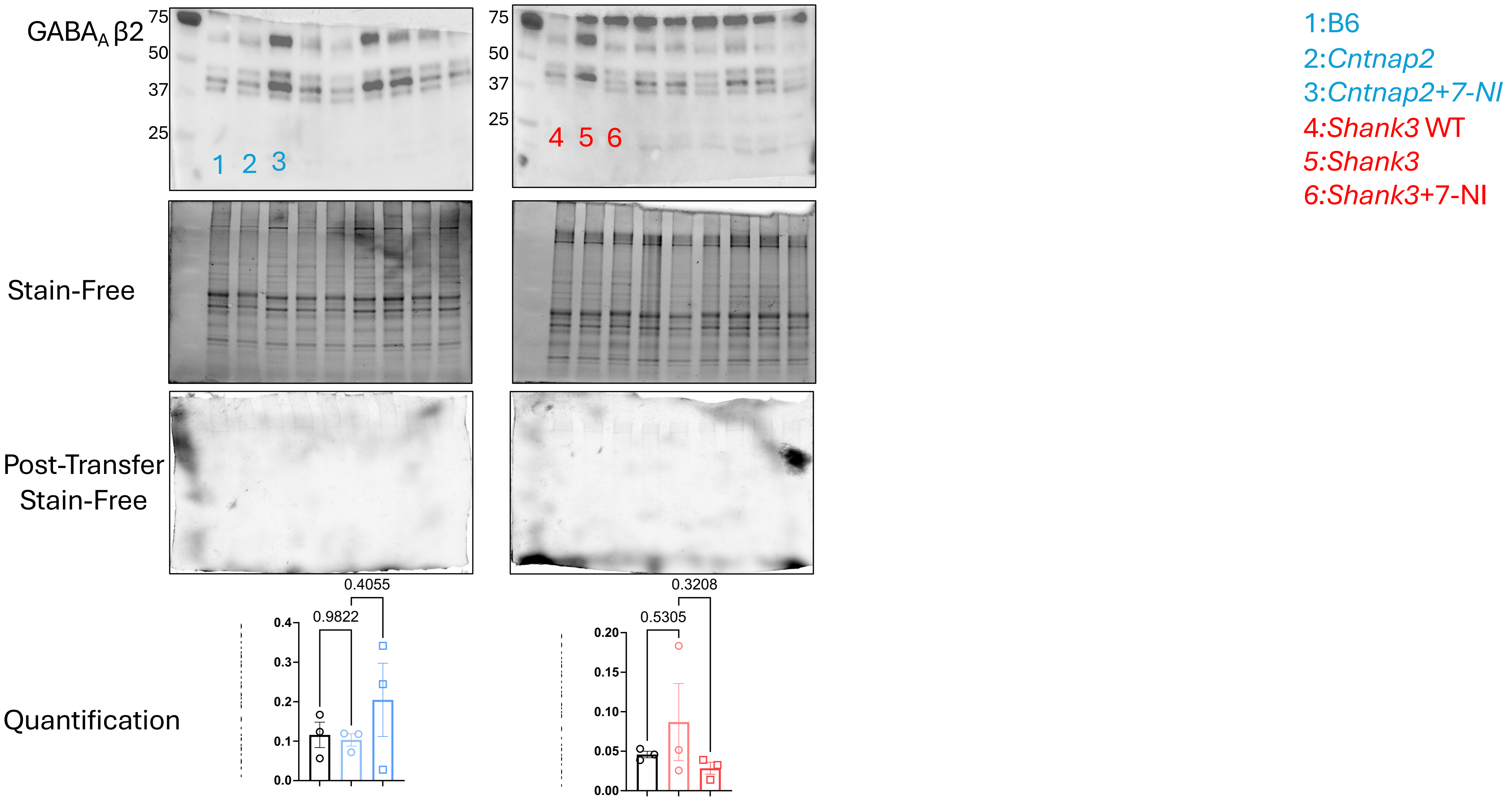

GABA$_A$ β2
75
50
37
25
1 2 3
4 5 6
Stain-Free
Post-Transfer Stain-Free
Quantification
0.4055
0.9822
0.3208
0.5305
0.4
0.3
0.2
0.1
0.0
0.20
0.15
0.10
0.05
0.00
1:B6
2:Cntnap2
3:Cntnap2+7-NI
4:Shank3 WT
5:Shank3
6:Shank3+7-NI

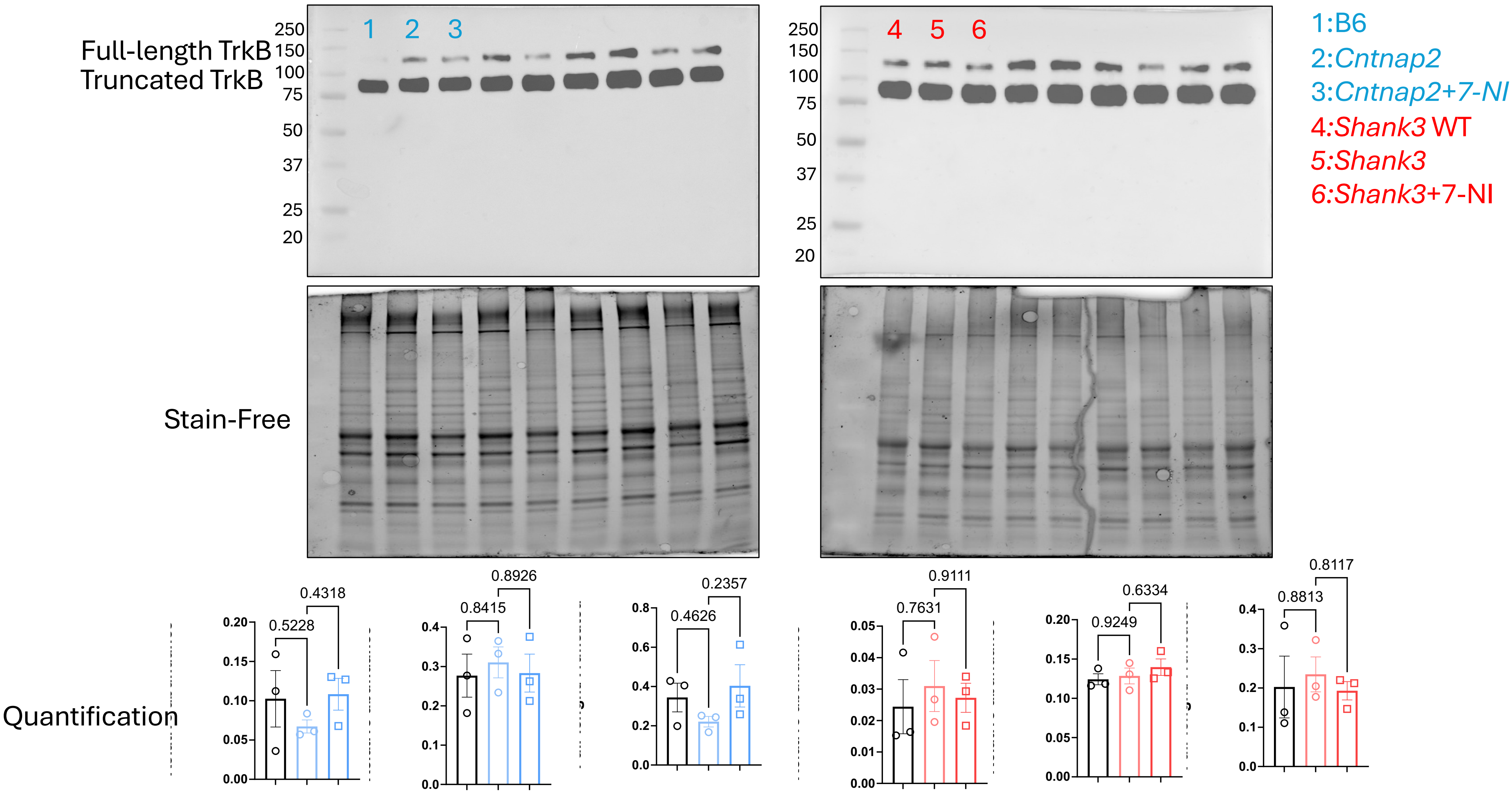

Full-length TrkB
Truncated TrkB
250
150
100
75
50
37
25
20
1
2
3
4
5
6
1:B6
2:*Cntnap2*
3:*Cntnap2+7-NI*
4:*Shank3* WT
5:*Shank3*
6:*Shank3*+7-NI
Stain-Free
Quantification
0.5228
0.4318
0.8415
0.8926
0.4626
0.2357
0.7631
0.9111
0.9249
0.6334
0.8813
0.8117
